# Reproductive concerns and associated factors among female chronic kidney diseases patients: a Multi‐Center Cross‐Sectional Study

**DOI:** 10.1002/nop2.850

**Published:** 2021-03-10

**Authors:** Fang Wang, Dengyan Ma, Stephen Salerno, Yi Chen, Min Liu, Yan Luo, Yongshu Diao, Chongcheng Chen, Ping Fu, Yi Li

**Affiliations:** ^1^ Department of Nephrology West China Hospital, Sichuan University/ West China School of Nursing, Sichuan University Chengdu Sichuan China; ^2^ Department of Biostatistics University of Michigan Ann Arbor MI USA

**Keywords:** Chronic kidney disease, factors, fertility intention, reproductive concerns

## Abstract

**Aim:**

The study aimed to investigate the current status of reproductive concerns and explore the associated factors among young female chronic kidney disease (CKD) patients.

**Design:**

A multi‐center cross‐sectional study was designed.

**Methods:**

The study was conducted in six representative tertiary hospitals across southwest China. A total of 295 female Chronic kidney disease patients between 18–45 years of age completed a 20 min, web‐based survey, which included demographics and disease‐related information questionnaire, Reproductive Concerns Scale, Generalized Anxiety Disorder 7 (GAD‐7) instrument and Patient Health Questionnaire 9 (PHQ‐9) instrument.

**Result:**

The survey total collected 270 valid questionnaires. The mean reproductive concern score was 54.39 ± 10.90 (out of a maximum of 90), with the mean scores for sub‐scales ranging from 7.80 ± 1.69 to 10.44 ± 1.85. Multiple regression analysis showed that those with higher reproductive concerns were more likely to have pregnancy intentions, to be in Chronic kidney disease stages 1–3, and to have a higher GAD‐7 score. This study offered further evidence of the need for improved education and emotional support surrounding reproductive concerns among young Chinese women with Chronic kidney disease.

## 1 INTRODUCTION

Chronic kidney disease (CKD) is a significant physical and economic burden all over the world. The prevalence of CKD was 10% in China (Bikbov et al., 2020), it was higher in China's southwest region (18.3%), and more than 50% of China's CKD patients were female (Wang, Yang, et al., 2019; Zhang et al., 2012). CKD patients could experience a series of physical symptoms, psychological problem, and changes in lifestyle (Lin et al., 2017). Further, as kidneys are important to be pregnancy, the associated risks of pregnancy increase with CKD severity (Hladunewich, 2017), so female CKD patients may have potential adverse pregnancy outcomes. By compared to women without CKD, female CKD patients had higher risk of adverse fetal and maternal outcomes (Barrett et al., 2020). Recent trends in couples delaying marriage, there is a greater prevalence of female CKD patients who have the intention to be pregnancy. Moreover, couples could have one more children in China, young women with CKD may consider having a second child after giving birth to one child (Wang et al., 2016). As women with CKD are aware of possible complications for their pregnancy, most of them are concerned with fertility after diagnosis.

Reproductive concerns have a significant influence on the quality of life for reproductive‐aged female cancer survivors, so the same with CKD patients. Particularly, concerns about infertility are common and may lead to emotional distress when diagnosed CKD. Reproductive concerns are not only common in young female cancer survivors (Ljungman et al., 2018), but also in female CKD patients. Reproductive concerns can last for a long time when diagnosed, and in some situations, may be more serious than the disease itself. Compared with female cancer survivors, young female CKD patients may face even greater anxiety surrounding fertility, causing greater psychological stress with infertility. The early identification of reproductive concerns is important to female CKD patients, as it can provide the basis for the treatment and infertility‐related counseling. So it is important to pay close attention to the reproductive concerns of young female CKD patients.

However, to our knowledge, we did not search specific scales to measure the signs of reproductive concern, and there are no studies to explore the associated factors of reproductive concerns in female CKD patients. The current body of literature on reproductive concerns almost pertains to cancer patients, there are specific scales such as the Reproductive Concerns After Cancer (RCAC) scale (Gorman et al., 2014). The RCAC has been sinicized and used on Chinese female cancer patients and has a suitable reliability and validity (Qiao et al., 2017). According to the previous studies, the associated factors of reproductive concerns included demographics, illness severity, anxiety and depression among cancer patients (Huang et al., 2020; Ljungman et al., 2018; Villarreal‐Garza et al., 2017). But little is known about the factors associated with reproductive concerns among CKD patients. Thus, the study used the Chinese version of RCAC scale to assess the symptoms of reproductive concerns among young female CKD patients and aimed to explore the factors related to reproductive concerns among these patients.

## 2 METHODS

### 2.1 Sample

This study was a multi‐center cross‐sectional survey and the sample included 295 patients at six representative tertiary hospitals in six cities across southwest China, who were enrolled between January–October 2019. Participants were included who met the following inclusion criteria: (a) diagnosed with CKD at least one year, (b) female, (c) aged between 18 and 45 years, (d) native Chinese and (e) informed consent. These participants were excluded: (a) currently pregnant, (b) with serious complication or diseases and (c) cognitive or behavioral impairment.

### 2.2 Data collection

Investigators were uniformly trained by the researchers. Before the survey, the investigators introduced the purpose of the study to participants, participants were informed that the study was conducted under the principles of anonymity and confidentiality, the patients voluntarily signed the informed consent form. The survey was administered in‐person through WeChat, a social media application, before or after the CKD management clinic visit. Each WeChat account could only submit one questionnaire. This would avoid the possibility of double registration.

### 2.3 Instruments

Participant demographics and disease‐related information were collected, including age, personal income, education level, marital status and employment status, disease staging, CKD period, number of children and intention to become pregnant (yes or no). In addition, the following three questionnaires were used to survey.

#### 2.3.1 Patient Health Questionnaire 9 (PHQ‐9)

The PHQ‐9 was designed to assess the symptoms of depression with a nine‐item (Kroenke et al., 2001). Each item ranged from 0 (none)–3 (nearly every day), the total score ranged from 0 to 27, with scores of 5, 10, 15 and 20 indicating mild, moderate, moderately severe, and severe depression, respectively. The Chinese version of PHQ‐9 has been validated and used as an efficient self‐administered screening tool to evaluate depression in China (Liu et al., 2016; Wang et al., 2014). Cronbach's α coefficient in this study was 0.91.

#### 2.3.2 Generalized anxiety disorder 7 (GAD‐7)

The GAD‐7 was a seven‐item, self‐administered tool designed to evaluate symptoms of anxiety (Spitzer et al., 2006). Individual components of the GAD‐7 were scored on a four‐point Likert scale, ranging from 0 (none) to 3 (nearly every day), with the total score for the instrument ranging from 0 to 21. The Chinese version of GAD‐7 has been used as a suitable screening instrument for detecting anxiety in China (Gong et al., 2020). Cronbach's α coefficient in this study was 0.87.

#### 2.3.3 The Chinese version of Reproductive Concerns After Cancer (RCAC)

RCAC was designed to assess fertility and parenthood concerns with an 18‐item for female cancer survivors (Gorman et al., 2014). Total scores ranged from 18 to 90 points with each item using a five‐point Likert. Six sub‐scales represent six dimensions of reproductive concerns. The RCAC scale has been validated and used as an efficient self‐reported tool to assess reproductive concern (Cronbach's α = 0.82) (Gorman et al., 2014). The Chinese version of RCAC has been proved to be a reliable and valid tool to evaluate the symptoms of reproductive concern among cancer patients, with the Cronbach's α coefficients ranged from 0.71 to 0.81 and the intra‐class correlation coefficients ranged from 0.82 to 0.95 (Qiao et al., 2017). The Chinese version of RCAC has not been used among female CKD patients. The study tested the validity and reliability of the Chinese version of RCAC to suitable for female CKD patients before this survey. In this study, the overall content validity index value was calculated to be 0.89, and item‐specific index values ranged from 0.75 to 0.96. The overall test–retest intra‐class correlation was 0.88 and ranged from 0.80 to 0.95 for the individual domains.

### 2.4 Data analysis

SPSS 22.0 software was performed for statistical analysis. Summary statistics for continuous variables were presented as the mean and standard deviation (*SD*), and categorical variables were reported as frequencies and percentages. Correlations between variables were assessed using Pearson's correlation when the data were jointly normal or Spearman's rank correlation when the data were not normal. One‐way analysis of variance was used for inter‐group comparisons, and multiple regression was used to test the independent risk factors for reproductive concerns among CKD patients. The level for statistical significance was set at .05.

## 3 RESULTS

### 3.1 Descriptive analysis of the participants

This study distributed 295 questionnaires, and 270 were returned, and the response rate was 91.5%. Thus, 270 patients were carried forward as the analytical sample. Missing values were avoided altogether by implementing a must‐answer design within the online questionnaire. The age of the participants were 18 to 45 years old (mean 53.62 ± 14.37 years). A majority were married (72.2%), had a college education (56.7%), were currently employed (55.2%), were diagnosed with CKD within five years (57.0%) and were in stage 1 to 3 (76.6%). Average monthly household income was more than 5,000 RMB (~$710) in 33.3% of participants. A minority reported having no intention of becoming pregnant (39.3%) and having no children currently (34.8%). Results were shown in Table 1.

**TABLE 1:** Patient characteristics for the study participants (*n* = 270)

Characteristic	Value
Gender, *n* (%)	
Female	270 (100.0)
Age (years)	
Mean (*SD*)	32.9 ± 7.1
Marital status, *n* (%)	
Single	63 (23.3)
Married	195 (72.2)
Divorced	12 (4.4)
Education level, *n* (%)	
Primary school or lower	13 (4.8)
Junior high school	54 (20.0)
Senior high school	50 (18.5)
University or above	156 (56.7)
Employment status, *n* (%)	
Employed	149 (55.2)
Unemployed	121 (44.8)
Personal income, *n* (%)	
1,000 RMB and below	52 (19.3)
1,000–2,999 RMB	56 (20.7)
3,000–4,999 RMB	72 (26.7)
≥5,000 RMB	90 (33.3)
Number of children, *n* (%)	
No children	102 (34.8)
One child	136 (50.4)
Two children	32 (11.9)
Fertility intention, *n* (%)	
Yes	106 (39.3)
No	164 (60.7)
Reasons for no fertility intention, *n* (%)	
Disease causes	54 (32.9)
Already have children	96 (58.5)
Others	14 (8.6)
CKD period since diagnosis, *n* (%)	
5 years and below	154 (57.0)
5–9 years	68 (25.2)
10 years and above	48 (17.8)
CKD stage, *n* (%)	
1	121 (44.8)
2	33 (12.2)
3	53 (19.6)
4	20 (7.4)
5	43 (15.9)

### 3.2 Reproductive concern among CKD patients

The mean reproductive health score in the study sample was found to be 54.39 ± 10.90, with the mean ranking scores of the six dimensions being as follows: personal health (10.44 ± 1.85), child health (9.82 ± 2.20), fertility potential (9.14 ± 1.78), becoming pregnant (9.10 ± 1.52), partner disclose (8.08 ± 1.67) and acceptance (7.80 ± 1.69). These results were shown in Table 2.

**TABLE 2:** Reproductive health scale outcomes for the *n* = 270 female CKD patients in the study sample

Subscale (Items)	Number of items	Mean ± *SD*	Ranking
Fertility potential	3	9.14 ± 1.78	3
Partner disclose	3	8.08 ± 1.67	5
Child's health	3	9.82 ± 2.20	2
Personal health	3	10.44 ± 1.85	1
Acceptance	3	7.80 ± 1.69	6
Becoming pregnant	3	9.10 ± 1.52	4
Overall scale	18	54.39 ± 10.90	/

### 3.3 Unadjusted results

As shown in Table 3, the reproductive health score was significantly associated with marital status (*p* < .001), education level (*p* = .011), employment status (*p* = .048), average monthly income (*p* < .001), number of children (*p* = .001), intention to become pregnant (*p* < .001) and CKD stage (*p* < .001). As shown in Table 4, the reproductive health score correlated with GAD‐7, PHQ‐9, and age. The reproductive health score was found to be positively correlated with GAD‐7 score (*p* < .001) and PHQ‐9 score (*p* < .001) and negatively correlated with age (*p* < .001).

**TABLE 3:** Univariate analysis of study variables.

Characteristic	Frequency (%)	Score (Mean ± *SD*)	*F/t*‐Value	*p*‐value
Marital status, *n* (%)				
Single	63 (23.3)	57.67 ± 10.44	**3.957**	**0.001**
Married	195 (72.2)	53.29 ± 10.78		
Divorced	12 (4.4)	55.08 ± 12.52		
Education level, *n* (%)				
Primary school or lower	13 (4.8)	47.23 ± 17.24	**3.815**	**.011**
Junior high school	54 (20.0)	51.81 ± 11.64		
Senior high school	50 (18.5)	54.88 ± 9.95		
University or above	153 (56.7)	55.75 ± 9.94		
Employment status, *n* (%)				
Employed	149 (55.2)	55.60 ± 9.73	**1.990**	**.048**
Unemployed	121 (44.8)	52.90 ± 12.06		
Personal income, *n* (%)				
1,000 RMB and below	52 (19.3)	49.06 ± 14.56	**6.688**	**0.001**
1,000–2,999 RMB	56 (20.7)	56.27 ± 9.21		
3,000–4,999 RMB	72 (26.7)	57.17 ± 9.16		
≥5,000 RMB and above	90 (33.3)	54.08 ± 9.63		
Number of children, *n* (%)				
No children	102 (34.8)	57.40 ± 10.84	**6.900**	**.001**
One child	136 (50.4)	52.90 ± 10.65		
Two children	32 (11.9)	51.13 ± 10.15		
Fertility intention, *n* (%)				
Yes	106 (39.3)	59.09 ± 9.57	**6.074**	**0.001**
No	164 (60.7)	51.35 ± 10.64		
CKD period since diagnosis, *n* (%)				
5 years and below	154 (57.0)	55.13 ± 10.98	0.839	.433
5–9 years	68 (25.2)	53.53 ± 9.74		
10 years and above	48 (17.8)	53.23 ± 12.13		
CKD stage, *n* (%)				
1–3	207 (76.7)	56.13 ± 9.63	**4.266**	**0.001**
4–5	33 (23.3)	48.68 ± 12.79		

Note

Frequencies presented as *n* (%) for *n* = 270 study participants. Scores are presented as mean ± *SD*, and requisite test statistics and *p*‐values are reported.

*P* values which are less than .05 are considered statistically significant and indicated in bold.

**TABLE 4:** Correlation results between reproductive concern score, psychological factors and age for *n* = 270 study participants

Item	*r*	*p*‐value
GAD‐7^a^	.283	<.001
PHQ‐9^a^	.281	<.001
Age^b^	−.280	<.001

Abbreviations: GAD‐7, 7‐item Generalized Anxiety Disorder Scale; PHQ‐9, 9‐item Patient Health Questionnaire.

^a^ Spearman.

^b^ Pearson.

### 3.4 Independent risk factors for reproductive concern

As shown in Table 5, in a multiple linear regression, reproductive health score was taken to be the dependent variable and statistically significant risk factors from the unadjusted analysis were taken as independent predictors. Multiple linear regression showed that intent to become pregnant (*p* < .001), CKD stage (*p* < .001) and GAD‐7 (*p* = .008) were all significant associated with reproductive health concern among the study participants.

**TABLE 5:** Multiple linear regression results for factors influencing reproductive health score among the *n* = 270 study participants

Factors	*B*‐value	*SE*	*β*‐value	*t*‐value	*p*‐value
Constant	71.504	6.623	/	10.796	<.001
Age	−0.155	0.112	−0.101	−1.378	.170
Marital status	−0.756	1.592	−0.034	−0.475	.635
Education	0.77	0.896	0.067	0.860	.391
Employment status	−0.878	1.508	−0.040	−0.583	.561
Average monthly income	−0.244	0.701	−0.025	−0.348	.728
Number of children	−0.497	1.146	−0.030	−0.433	.665
Fertility intention	−5.168	1.375	−0.232	−3.758	<.001
CKD stage	−4.680	1.548	−0.182	−3.024	<.001
GAD‐7	0.537	0.202	0.247	2.658	.008
PHQ‐9	0.020	0.183	0.010	0.109	.914

Note

Adjust *R* ^2^ = .250; *F* = 8.622; *p* < .001.

Abbreviations: *B*, Unstandardized coefficients; *p*, *p*‐value; *SE*, Standard error; *t*, *t*‐statistic; *β*, Standardized coefficients.

## 4 DISCUSSION

Identifying concerns related to fertility and parenthood is essential for meeting the long‐term reproductive health goals of young female CKD patients. This study was the first evaluation of reproductive concerns among young female CKD patients using the Chinese version of RCAC in China. The findings of this study showed that intent to become pregnant, CKD stage, and the GAD‐7 score were significant factors associated with reproductive concerns among these patients. This offered further evidence of the need for improved education and emotional support surrounding reproductive concerns among young Chinese women with CKD.

In the study, the average reproductive concern score for CKD patients was found to be lower than what was reported for Chinese female cancer patients (Wang, Cheng, et al., 2019; Yuan & Zheng, 2018). This could be due to the severity of the cancer disease, and the other possible explanation was that ovarian germ cells were damaged by the cancer treatments including radiation or chemotherapy, this could lead to a series of reproductive problems, such as sterility or diminished fertility (Cvancarova et al., 2009; Hamre et al., 2012). In our study, female CKD patients felt concerned about fertility if they received inadequate or conflicting reproductive information or if they were uncertain about their fertility status. This study further indicated that CKD patients had a highly awareness of the importance of fertility. The ranking scores of six dimensions showed that CKD patients had important concerns about personal and child health issues that extended beyond other concerns. Each sub‐scale represented a type of concern which could be addressed clinically to promote health well‐being and quality of life for a long time, particularly with respect to personal and child health issues. The findings of our studies also indicated that the reproductive concerns of young female CKD patients had a diverse range, as this was the first study to investigate these concerns among young female CKD patients in China.

Our regression analysis showed that CKD patients with more reproductive concerns were more likely to want to become pregnant, were in CKD stages 1–3 versus 4–5, and had higher GAD‐7 scores. Previous studies have also showed that reproductive concerns were higher among young adult female cancer survivors who reported wanting to have a baby (Gorman et al., 2014; Shah et al., 2016). In our study, women in stages 1–3 were similarly likely to voice concerns that their illness may affect their ability to have children, while most women in stages 4–5 did not intend to have children because of the seriousness of their disease. Total reproductive concerns scores were also positively correlated with the GAD‐7 and PHQ‐9 instruments, consistent with previous studies (Gorman et al., 2010, 2014, 2015; Qiao et al., 2017). This pattern of association further supported the validity of the reproductive concern scale for young female CKD patients. In addressing these issues, healthcare providers should get additional training and skill to handle these sensitive topics and solve reproductive problems for their patients (Stark et al., 2019). As a result, female CKD patients should be educated about their reproductive status and potential.

## 5 CONCLUSIONS

In conclusion, this study showed female CKD patients had a different level of reproductive concerns status. Education and emotional support are needed to improve the reproductive concerns in these patients, especially in patients with pregnancy intentions, CKD stages 1–3, and a higher GAD‐7 scores.

### 5.1 Relevance to clinical practice

Identifying such concerns is important to improve the reproductive health and long‐term quality of life for young female CKD patients. Healthcare providers could benefit from additional training in handling these sensitive topics and improved education regarding reproductive concern among their patients. Moreover, young women should have access to counseling service and should be made informed decisions about their fertility problems (Letourneau et al., 2012; Young et al., 2019). Young female patients who have reproductive concerns should early be referred to a reproductive health specialist who may improve the reproductive quality (Shah et al., 2016). Therefore, it is very essential to consider the reproductive health of young female CKD patients in China and adopt personalized strategies to improve their reproductive health.

### 5.2 Limitations

This study has a few limitations. First, the study was a cross‐sectional design that limited to draw any causal conclusions. Future prospective longitudinal studies are needed to explore the fertility‐related services for the young female CKD patients. Second, the sample only represented the CKD patients of the southwest China, sample size needs to be enlarged all over China. Additional, qualitative research can be designed to further explore the factors of reproductive concerns among young female and male CKD patients.

## CONFLICT OF INTEREST

The authors declare that they have no conflict of interest.

## AUTHOR CONTRIBUTIONS

Fang Wang and Dengyan Ma designed the study and developed the analytical strategy. Yi Chen, Min Liu, Yan Luo and Chongcheng Chen collected the data. Dengyan Ma and Fang Wang performed statistical analysis and drafted the manuscript. Dengyan Ma, Salerno Stephen, Yongshu Diao, Ping Fu and Yi Li critically revised the manuscript. All authors have read and approved the final manuscript.

## ETHICAL APPROVAL

This study was approved by the human research ethics committee of the corresponding institution. And complied with the STROBE checklist (File S1). The online survey instrument for the study was anonymous. Each participant was voluntary, informed consent was obtained when they accessed the online survey.

## Data Availability

The data that support the findings of this study are available from the corresponding author upon reasonable request.
